# Synaptic migration and reorganization after noise exposure suggests regeneration in a mature mammalian cochlea

**DOI:** 10.1038/s41598-020-76553-w

**Published:** 2020-11-17

**Authors:** Tyler T. Hickman, Ken Hashimoto, Leslie D. Liberman, M. Charles Liberman

**Affiliations:** 1 Eaton-Peabody Laboratories, Massachusetts Eye and Ear, 243 Charles St., Boston, MA 02114-3096 USA; 2 Department of Otolaryngology-Head and Neck Surgery, Harvard Medical School, Boston, MA 02115 USA; 3 Department of Otorhinolaryngology, Tohoku University School of Medicine, Sendai, Japan

**Keywords:** Neurophysiology, Peripheral nervous system, Neurodegeneration, Peripheral neuropathies

## Abstract

Overexposure to intense noise can destroy the synapses between auditory nerve fibers and their hair cell targets without destroying the hair cells themselves. In adult mice, this synaptopathy is immediate and largely irreversible, whereas, in guinea pigs, counts of immunostained synaptic puncta can recover with increasing post-exposure survival. Here, we asked whether this recovery simply reflects changes in synaptic immunostaining, or whether there is actual retraction and extension of neurites and/or synaptogenesis. Analysis of the numbers, sizes and spatial distribution of pre- and post-synaptic markers on cochlear inner hair cells, in guinea pigs surviving from 1 day to 6 months after a synaptopathic exposure, shows dramatic synaptic re-organization during the recovery period in which synapse counts recover from 16 to 91% of normal in the most affected regions. Synaptic puncta move all over the hair cell membrane during recovery, translocating far from their normal positions at the basolateral pole, and auditory-nerve terminals extend towards the hair cell’s apical end to re-establish contact with them. These observations provide stronger evidence for spontaneous neural regeneration in a mature mammalian cochlea than can be inferred from synaptic counts alone.

## Introduction

Acoustic overstimulation causes hearing impairment by permanently damaging the delicate sensory structures of the inner ear. The hair cells, in particular, are vulnerable to acoustic injury^1^, especially the outer hair cells (OHCs), which function as biological amplifiers of the sound-evoked motions of the sensory epithelium^2^. Thus, OHC degeneration causes elevation of cochlear thresholds, i.e. the hearing loss measured behaviorally by the audiogram. Since hair cell death is irreversible in the mammalian inner ear^3^, noise-induced hearing loss often has a permanent component.

Hair cells, however, are not the most vulnerable elements to acoustic overexposure^4,5^. Even exposures causing only transient threshold elevation, and thus no hair cell death, can destroy synaptic connections between auditory nerve fibers (ANFs) and inner hair cells (IHCs). These synaptic connections normally drive the fast signal transmission required for encoding of acoustic signals^6^. This synaptopathy does not significantly affect simple auditory tasks, like threshold detection, until it is nearly complete^7^, but it likely affects discrimination of complex sounds, especially in noisy environments. It has also been implicated in the generation of tinnitus and hyperacusis, two poorly understood comorbidities of noise-induced hearing loss^8,9^.

In mice, noise-induced loss of up to 50% of ANF synapses can be seen immediately after exposure in IHCs immunostained for pre- and post-synaptic markers^10^. The phenomenon, assumed to reflect a type of glutamate excitotoxicity, was initially observed at the electron microscopic level, where acutely noise-exposed ears showed swelling and rupture of ANF terminals underneath the IHCs^11,12^. This pathology was assumed to be reversible, since cochlear thresholds could return to normal^13^. However, confocal studies in CBA/CaJ mice suggested that the noise-induced loss of synaptic puncta is irreversible. Indeed, when evaluated at long post-exposure times, loss of ANF cell bodies ultimately matched the immediate 50% loss of ANF/IHC synapses^5,14,15^.

Subsequent confocal studies in noise-exposed guinea pigs also reported a massive loss of pre- and post-synaptic puncta, without hair cell degeneration, in the days immediately post exposure, but observed almost full post-exposure recovery of immunopositive puncta, and concluded there was widespread regeneration of ANF terminals^16^. However, in our studies of noise-exposed mice, immunostaining intensity of surviving synaptic puncta is often transiently reduced by trauma. Thus, the disappearance and reappearance of synaptic puncta might reflect a noise-induced down- and up-regulation of synaptic protein expression, such that the immunohistochemical signals are transiently below detection levels. Furthermore, prior guinea pig regeneration studies analyzed presynaptic and postsynaptic immunomarkers independently, making it unclear whether the reappearing elements were closely apposed as in normal synaptic configurations.

Our aim was to clarify whether this noise-induced decrease and increase in synaptic puncta counts in guinea pig reflects a recovery process or a regeneration process, i.e. whether destruction and reconstruction of ANF synapses really occurs after noise exposure in an adult mammalian ear. Our approach was to supplement the simple 2-D counts of synaptic puncta, as done in prior studies, with an analysis of size and 3-D spatial organization of the pre-synaptic ribbons, and to add glutamate-receptor labeling and dendritic markers to directly visualize the ANF terminals and their synapses. Results show that synapses in the guinea pig cochlea are moving around the IHC membrane after exposure, and that ANFs are extending neurites that form synaptic connections during the recovery process. We speculate that the discrepant results between mouse and guinea pig could be related to differences in age-at-exposure, as well as differences in species or strain.

## Methods

### Subjects and groups

Female Hartley guinea pigs, at ~ 250 g, were used in this study. Birthdates are unavailable for our animals, but our supplier’s website (elmhilllabs.com) states that their guinea pigs are 2–3 weeks old at that weight. After a 7-day acclimation to the animal care facility, unanesthetized animals were placed in a wire-mesh cage on a rotating platform within a reverberant chamber, exposed to noise (4–8 kHz, 106 dB SPL, 2 h), and allowed to recover for 1 day, 1 week, 1 month, 2 months or 6 months before cochlear function tests, i.e. CAPs (compound action potentials) and DPOAEs (distortion product otoacoustic emissions), immediately followed by tissue harvest. There were at least four animals in each group. One group of four males was similarly exposed and allowed to survive for one month. Unexposed controls were housed with exposed animals of the same age. The exposure was designed to induce large threshold shifts with minimal hair cell loss. All procedures were approved by the IACUC of the Massachusetts Eye and Ear, and were performed in accordance with the “Guide for Care and Use of Laboratory Animals."

### Cochlear function

Cochlear function tests were carried out in an electrically shielded and sound-attenuating chamber, with ambient temperature maintained at 32 °C. Animals were deeply anesthetized with ketamine (100 mg/kg) and xylazine (8 mg/kg). Acoustic stimuli were delivered through a closed acoustic system, consisting of two sound sources (CDMG15008- 03A, CUI) and an electret condenser microphone (FG-23329-PO7, Knowles) as an in-dwelling probe microphone. Stimuli for CAPs were 4-ms tone pips (0.5 ms rise-fall, 19/sec repetition rate). CAP responses were recorded with a silver ball electrode on the round window membrane, referred to a ground in the neck muscle. Threshold was defined as the sound level, at each frequency, required to produce a response of 5 μV peak to peak, as determined by a computer-driven algorithm. DPOAEs were recorded in response to a pair of pure tones (f_1_ and f_2_), in a frequency ratio (f_2_/f_1_) of 1.2, and a level separation of 10 dB (f_1_ level > f_2_ level). DPOAE threshold was defined as the level of f_2_ required to produce a distortion product in the ear canal of 10 dB SPL, as determined by interpolation between 5 dB level steps at each f_2_ frequency.

### Tissue fixation, immunostaining and image acquisition

Animals were deeply anesthetized with fatal-plus solution (300 mg/kg pentobarbital sodium), and then tissue was fixed by intravascular perfusion of 4% paraformaldehyde following a saline wash-out; cochleae were flushed through the scalae with the same fix and then post-fixed for 2 h, decalcified for 2–3 weeks in 0.12 M EDTA, and dissected into roughly 11 pieces, each containing a fraction of the spiraling sensory epithelium. Tissue was permeabilized by freezing on dry ice in 30% sucrose and blocked for 1 h at 22 ºC in PBS with 1% Triton X + 5% normal horse serum. Tissue was then incubated overnight at 37 ºC in the following primary antibodies, diluted in 1% normal horse serum and Triton X in PBS: (1) mouse isotype IgG1 anti-C-terminal binding protein 2 (CtBP2, 1:50, BD Transduction Laboratories #612044), (2) mouse isotype IgG2 anti-glutamate receptor 2 (GluA2a, 1:1000, Millipore #MAB397), (3) rabbit anti-myosin VIIa (Myo7a, 1:100, Proteus BioSciences #25-6790), and (4) mouse anti-neurofilament H (NFH, 1:500, Millipore #AB5539). After washing in PBS, the tissue was incubated twice for 1 h each in the following secondaries, diluted in 1% normal horse serum and Triton X in PBS: (1) Alexa Fluor 568 goat anti-mouse IgG1 (1:1000, Thermo Fisher #A21124), (2) Alexa Fluor 488 goat anti-mouse IgG2a (1:500, Thermo Fisher #A21131), (3) Alexa Fluor 647 goat anti-chicken (1:200, Thermo Fisher #A21449), and (4) PacificBlue goat anti-rabbit (1:200, Thermo Fisher P10994).

Low-power images of the myosin channel in each microdissected piece were obtained with a 10× air objective (N.A. 0.4) on a Leica DM5500 epifluorescence microscope at sufficient resolution to count hair cells. Cytocochleograms were constructed from these images by tracing the cochlear spiral and superimposing hash marks at each 2% length increment using a custom ImageJ plugin^17^. Frequency correlates were also superimposed on the microdissected spiral by application of the cochlear frequency map for guinea pig^18^, and the organ of Corti was imaged at half- to one-octave intervals with a Leica confocal microscope using a 63× glycerol immersion objective (N.A. 1.3). At each of the desired locations (11 cochlear regions at log-spaced intervals from 0.5 to 45.25 kHz), in each case, two adjacent microscopic fields (9–12 IHCs per field) were imaged with a 4-channel z-stack spanning the height of the hair cells to faithfully capture all synaptic puncta in that region of interest (1024 × 512 pixels in x and y, at roughly 80 nm per pixel with z- spacing at 0.33 μm per slice).

### Image analysis

The numbers and volumes of ribbons were analyzed with Amira software (v 6.4, Visage Imaging) using the *connected components* feature to automate the identification of puncta in three-dimensional space, and the notation of their locations, sizes and numbers. The pairing of pre-synaptic ribbons with post-synaptic receptors and/or auditory nerve terminals was assessed with custom C++ software that re-projected the voxel space immediately around each ribbon (i.e. within 1 micron for CtBP2 and GluA2 analysis, and within 2 microns for CtBP2 and NFH analysis) as an array of thumbnail images, from which an observer can evaluate the postsynaptic staining around each ribbon: see Fig. 3 in a prior report from our lab^19^ for further details. The x,y,z coordinates of ribbons in each z-stack were transformed into a coordinate system based on the modiolar-pillar polarity of the IHCs, using a custom LabVIEW program. This program required user input only to define the line bisecting the subnuclear portion of the IHCs into pillar vs. modiolar halves, as seen in the maximum zy projection of each z-stack. This bisector became the transformed y axis; an x axis was created perpendicular to it, and placed such that the ribbons farthest from the cuticular plate were at y = 0. This manual operation was performed by an observer blinded to the exposure-group/survival-time information on each ear. See text for further details.

The HC height was defined as the distance from the middle of the cuticular plate to the middle of the habenular pole of the HC. The ANF height was defined as the distance—μm along this midline—that NFH antibodies stain an ANF. These measurements were made using ImageJ 1.52p (https://imagej.nih.gov/ij) and Photoshop CS6 (adobe.com).

### Statistics methodology

All statistical comparisons were made using Graphpad Prism v8. ANOVAs were adjusted for missing values with Prism’s mixed-effects-model 2-way ANOVA that calculates a compound symmetry covariance matrix, fitted using restricted maximum likelihood. This method accounts for rare, random missing values, which occur, for example, when a frequency place in a particular cochlea is damaged during processing. Within the mixed-effects-model, fixed effects were defined as frequency and recovery time, while random effects were defined as subjects (ears) and residuals. These tests allow more flexibility with fewer assumptions than 2-way repeated-measures ANOVAs, but calculate the same p values when there are no missing values. The Geisser-Greenhouse correction method was used to account for unequal variance between repeated measures, and D'Agostino-Pearson’s omnibus K2 normality tests were used to verify the normality of the data. Post hoc multiple comparisons were calculated using the Holm-Sidak multiple comparisons method.

## Results

### Threshold shifts and hair cell loss

**Figure 1:**
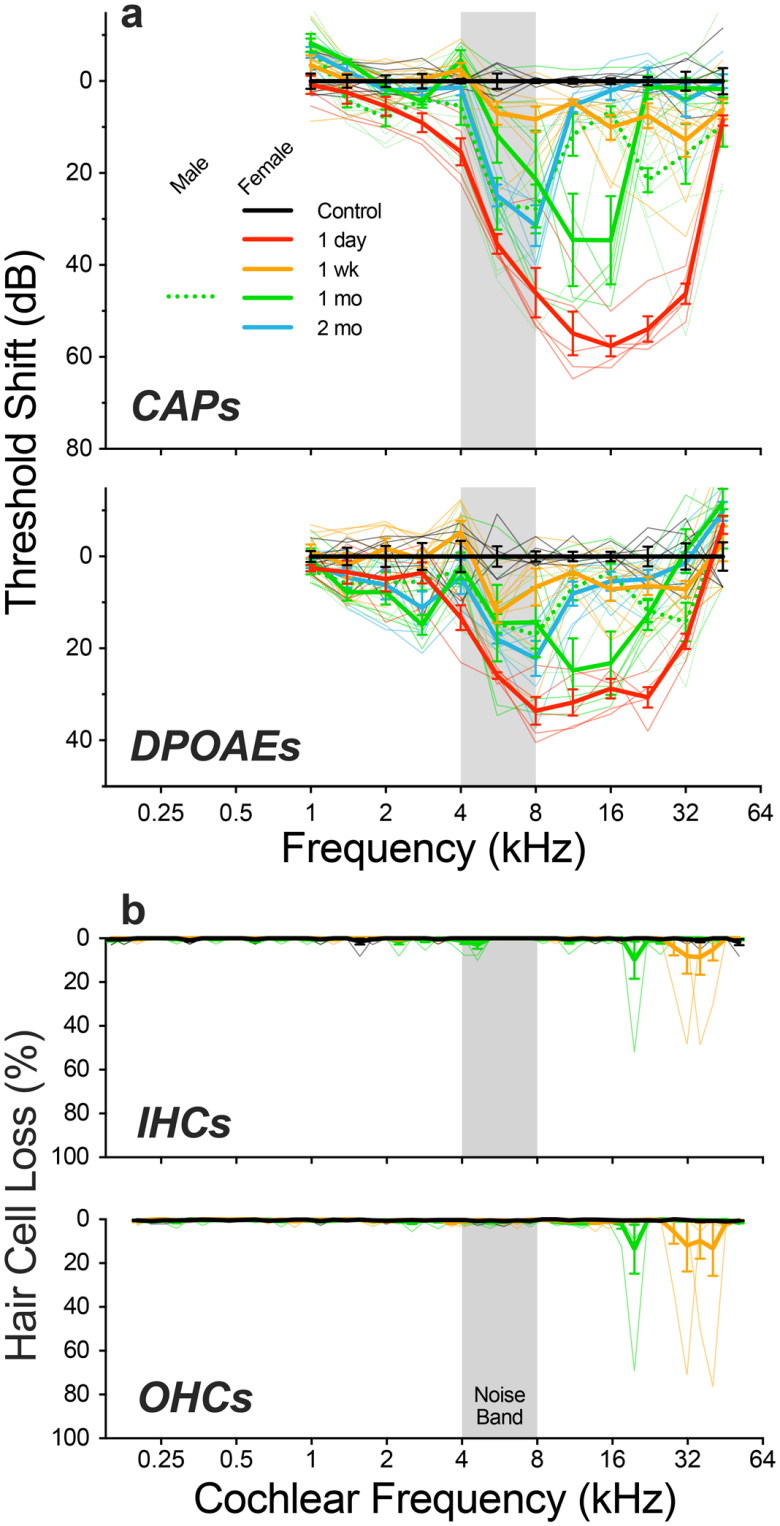
Exposure to octave-band noise produces a large TTS with minimal HC loss, from 1 day to 2 months post exposure. (**a**) Mean group values (thick lines) and single-ear values (thin lines) of threshold shift (± SEMs), as measured by CAPs or DPOAEs, at the post-exposure survival times indicated in the key. Group sizes for cochlear function tests were: *Control* (n = 6 ears, 3 animals), *1 day* (n = 5 ears, 3 animals), *1 week* (n = 9 ears, 5 animals), *1 month female* (n = 5 ears, 3 animals), *1 month male* (n = 8 ears, 4 animals), *2 months* (n = 6 ears, 3 animals). (**b**) Mean group values (thick lines) and single-ear values (thin lines) of inner and outer hair cell loss (± SEMs) seen in controls, 1-week, and 1-month post-exposure survival times. Group sizes for hair cell counts were: *Control* (n = 6 ears, 5 animals), *1 week* (n = 6 ears, 5 animals), *1 month* (n = 6 ears, 3 animals). The grey bar shows the frequency band of the acoustic overexposure. Cochlear function was not interpretable at 6 months, because many of the animals had developed middle-ear infections.

A 2-h exposure to octave-band noise at 106 dB SPL produced a large threshold elevation, when measured 1-day post-exposure (Fig. 1a). The peak threshold shift occurred at frequencies above the exposure band, as expected based on cochlear mechanical non-linearities^20^. Because of these non-linearities, the cochlear region responding maximally to 4 kHz at low sound pressures (which defines cochlear frequency map) is apical to the location vibrating maximally to 4 kHz at high levels, e.g. the 106-dB noise used here to produce acoustic injury.

In the group evaluated at 1 week post exposure, thresholds had recovered to within 13 dB of baseline measures (Fig. 1b), whether measured by CAPs (a metric of ANF responses) or DPOAEs (a metric of OHC amplifier function). The groups recovering for 1 month and 2 months showed larger threshold shifts. This post-exposure trend might reflect long-term deterioration after initial recovery, however the inter-animal variability in noise-induced hair cell damage in guinea pigs and other outbred animals is so large that no strong conclusion is possible^21^. Interestingly, the intragroup variability in histological assessment of synaptic damage was much lower (see below). To evaluate effects of sex on the noise vulnerability in this animal model, we included one group of males, studied at the 1-month time point: the magnitude of threshold shift was not significantly different (Fig. 1a; p = 0.35, 2-way ANOVA).

Based on prior work in cat, mouse, and guinea pig^22–24^, noise-induced PTS of this magnitude should not be associated with much hair cell loss, because the functionally important structural changes are to the stereocilia bundles of surviving cells. Indeed, the loss of inner and outer hair cells was minimal and rarely distinguishable from controls (Fig. 1b). The degeneration tended to be focal in nature, and was restricted to the extreme base of the cochlea, where noise-induced lesions often first appear^1^. At each post-exposure time, the similarity between the pattern of threshold shift measured by CAP vs DPOAE is consistent with the speculation that the functionally important damage is to the OHC stereocilia^22^.

### Synaptic counts as a function of post-exposure time

**Figure 2:**
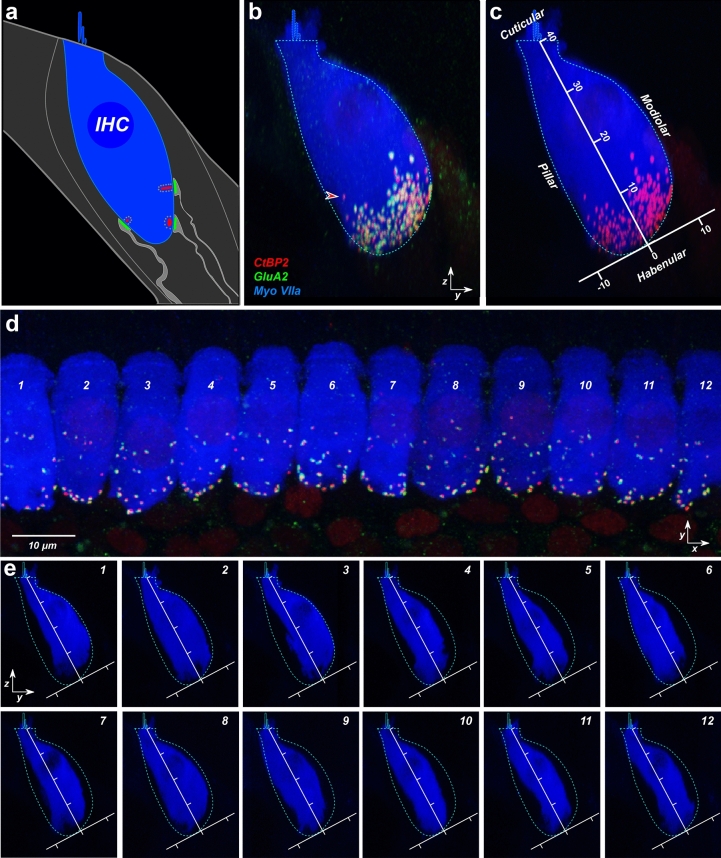
Synaptic locations were transformed to a hair-cell-based coordinate system. (**a**) Schematic shows the synapses of three auditory-nerve terminals (light gray, green, and red) around the basolateral surface of an IHC (blue). (**b**) A zy maximum projection of the image stack shown in (**d**) superimposes 12 adjacent IHCs from the 16-kHz region of a control ear. Arrowhead points to an orphan ribbon, i.e. one that is unpaired with a GluA2-positive punctum. The cyan, dashed shape superimposed on panels in (**b,c**,**e**) is an outline of the Myo7a-staining from the zy projection in panel (**b**). (**c**) To quantify ribbon sizes and positions across z stacks, we transformed the zy plane as schematized, placing a new vertical axis parallel to the IHCs’ long axis and bisecting the IHCs’ basolateral pole into pillar- and modiolar-facing halves. Axis values in **c** are in microns, and the image is the same as in (**b**), except the GluA2 channel is masked. (**e**) A maximum projection of each IHC in (**d**) is extracted from the z-stack and shown in the zy projection. Axes are the same as in (**c**).

As schematized in Fig. 2a, each ANF typically forms a single synaptic contact with a single IHC, via a terminal bouton with a post-synaptic membrane patch expressing glutamate receptors, positioned opposite a pre-synaptic ribbon in the IHC^25,26^. Thus, the number of synapses in the sensory epithelium can be accurately assessed by counting closely juxtaposed CtBP2- and GluA2-positive puncta in confocal image stacks such as the one in Fig. 2d.

**Figure 3:**
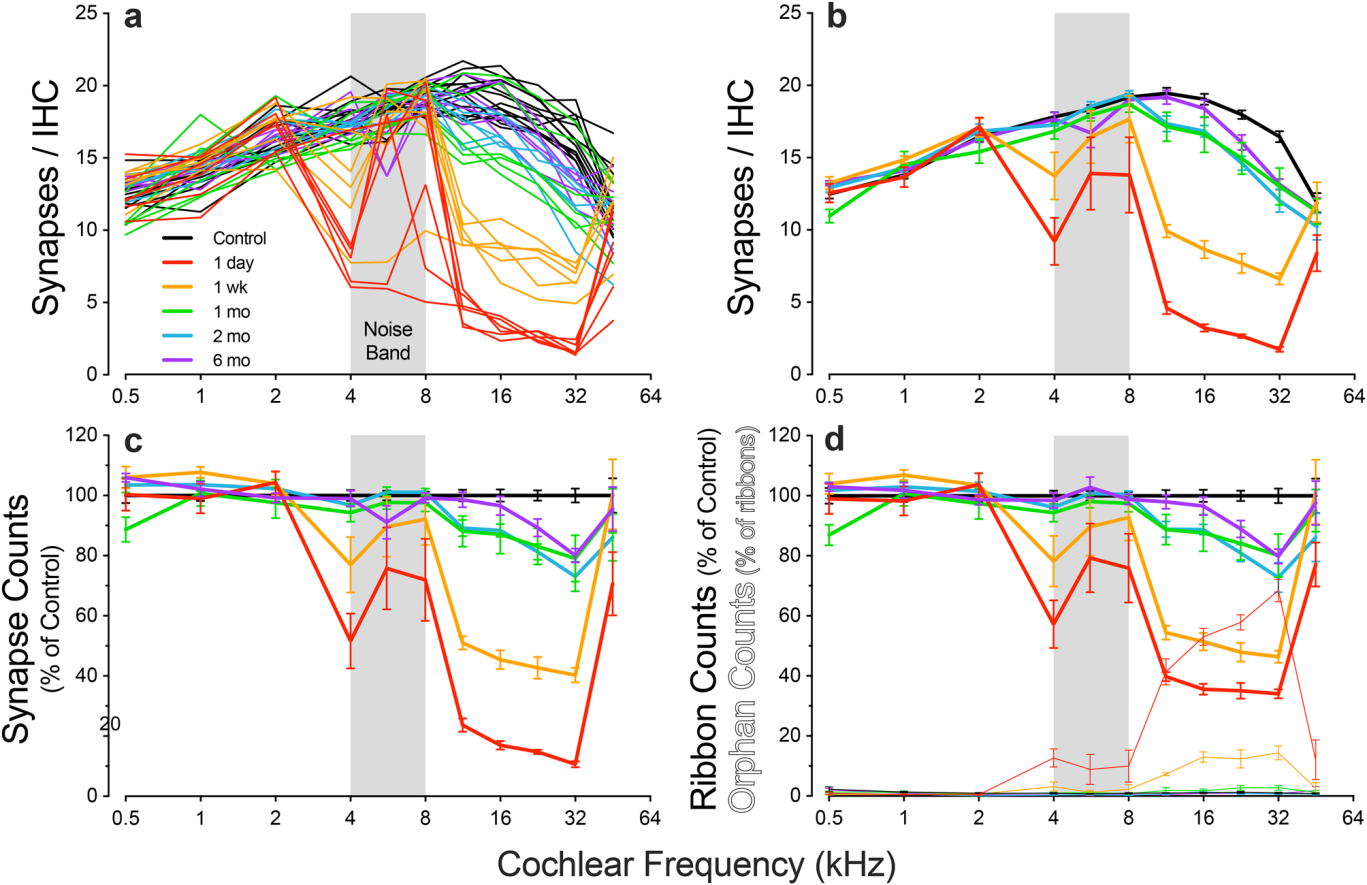
Synaptic puncta disappear then recover from 1 day to 6 months post exposure. **a,b**: Individual cases (**a**) and mean group values (**b**; ± SEMs) of synaptic counts per IHC in each of the post-exposure groups as indicated in the key. **c,d**: Mean survival (± SEMs) of synapses (**c**), i.e. CtBP2 puncta paired with GluA2 patches, or ribbons (**d**), total vs. orphan ribbons, i.e. all CtBP2 puncta (thick lines) vs. unpaired CtBP2 puncta (thin lines). Fractional survival is computed by normalizing counts to controls. Group sizes were as follows: *Control* (n = 13 ears, 7 animals), *1 day* (n = 6 ears, 3 animals), *1 week* (n = 6 ears, 5 animals), *1 month* (n = 6 ears, 3 animals), *2 months* (n = 6 ears, 3 animals), *6 months* (n = 6 ears, 3 animals).

Based on such counts, control ears show a broad peak of roughly 20 ANF synapses per IHC at cochlear regions tuned to frequencies between 8 and 16 kHz (Fig. 3a,b). That number decreases slowly to < 15 synapses per IHC towards the cochlear apex and more rapidly towards the cochlear base. As expected, threshold shifts and synapse loss are not closely linked; threshold shifts are likely dominated by stereocilia damage^22^, whereas even synapse losses in excess of 50% do not alter thresholds^5^.

One day post exposure, there is a substantial loss of paired synaptic puncta, spanning all cochlear regions from the 4-kHz locus to the base, with the loss peaking at 89% at 32 kHz (Fig. 3c). With increasing post-exposure time, the synaptic counts recover dramatically. By six months, the synaptic loss has decreased to a maximum of 20% at 32 kHz (Fig. 3c), though this is still significantly lower than controls (p = 0.0004, Holm-Sidak multiple comparisons test). Except for the 1- vs. 2-month post-exposure times, which are statistically indistinguishable (p = 0.67 for Group and p = 0.28 for Group x Frequency), all the other survival groups are significantly different from each other, either by comparison between Groups (when synaptopathy spreads over much of the cochlea, i.e. 1 day vs. 1 week, p = 0.003) or by the Group x Frequency interaction, when the synaptopathy is more restricted in extent, i.e. 1 week vs. 1 month, p < 0.0001 and 2 month vs. 6 months, p = 0.006). This stands in stark contrast to our laboratory’s data from CBA/CaJ mouse, where synaptic counts show only further decreases as post-exposure survival increases from 1 day to 2 years^5,14,15^.

During the first week post exposure, as reported in prior studies of synaptopathy^10^, many of the synaptic counts are lower than the ribbon counts, because many of the remaining pre-synaptic ribbons are “orphans,” unpaired with post-synaptic glutamate receptor patches (Fig. 3d), e.g. 68.5% of the surviving ribbons in the 32-kHz region were unpaired 1 day post-exposure, compared to only 1.1% in Controls. However, by 1 month, orphan ribbons had fallen to 2.7% and then further to 0.7% and 0.8% at 2 and 6 months, respectively. Across all tested frequencies, group differences in orphan ribbon counts at 1 day and 1 week vs. control are all statistically significant (p < 0.0001 for each), whereas the differences between 1, 2, or 6 months vs. control are not (p = 0.370, p = 0.120, and p = 0.437, respectively).

### Noise-induced changes in synaptic morphology and spatial organization

Most prior studies designed to track the post-exposure recovery of cochlear synapses after noise exposure assessed only puncta counts, of the type shown in Fig. 3, at a number of cochlear locations^5,16,27–30^. The decrease and subsequent increase in these counts, which is also seen here, could reflect either (1) a recovery process where, for example, the staining intensity of synaptic puncta in intact ANF terminals falls below detectability and then recovers, or (2) a regenerative process wherein the unmyelinated ANF dendrites within the sensory epithelium retract and regrow to re-establish new synaptic connections with the IHC. To help distinguish between these two fundamentally different interpretations, we evaluated the morphology and spatial organization of the synaptic puncta, and immunostained for the ANF dendrites themselves.

Electrophysiological studies have shown that ANFs can be functionally subdivided into at least two subgroups that differ in threshold sensitivity and spontaneous discharge rate (SR)^18,31,32^. In cat and guinea pig, single-fiber labeling studies have shown that the synapses of low- vs. high-SR ANFs are spatially separated onto the modiolar vs. pillar sides of the IHC, respectively^18,31^, and (somewhat paradoxically) are associated with larger vs. smaller pre-synaptic ribbons, respectively^33^. This gradient of ribbon size is easily seen in confocal z-stacks from normal ears, when they are re-projected onto the zy plane (Fig. 2b,c). The normal spatial restriction of these synapses to a U-shaped, sub-nuclear cluster conforming to the basolateral poles of the IHCs is also clearly seen in these re-projected images (Fig. 2b).

To systematically evaluate the noise-induced changes in these ribbon size gradients and ribbon localization patterns, we defined a modiolar-pillar axis for each z-stack based on the zy maximum projection (Fig. 2c), with the new y axis bisecting the subnuclear region of IHCs, running parallel to the long axis of the superimposed IHCs. Compared to the mouse cochlea^34^, the basolateral poles of IHCs in guinea pig are relatively well aligned: thus, the axis defined using the superimposed silhouette of 12 adjacent IHCs (e.g. Fig. 2c) does a more than adequate job of separating modiolar vs. pillar sides of each IHC in the stack (Fig. 2e). Given that the long axis of the IHC is often curved in the yz plane (e.g. cells 7, 9 and 11 in Fig. 2e), no straight line could “perfectly” divide the basolateral pole, even if a new coordinate system was defined for each single-cell projection.

**Figure 4:**
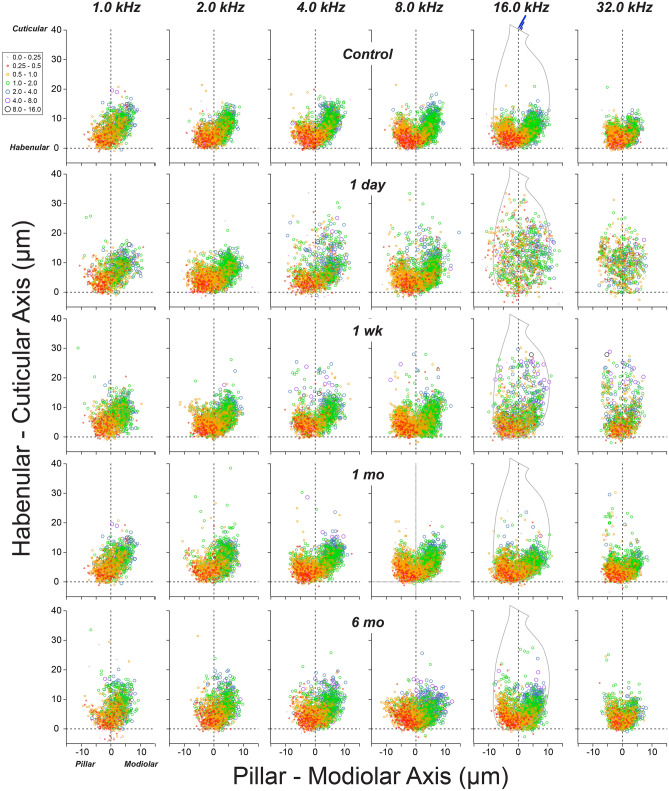
Post-exposure, IHC synaptic architecture becomes disorganized, and gradually reorganizes. Panels are organized into columns by cochlear location (i.e. frequency), and into rows by post-exposure survival. In each panel, the locations of synaptic ribbons are shown along hair-cell-relative axes (see Fig. 2 and dashed outline of 16 kHz controls for orientation): ribbon size is indicated by color and symbol size, as indicated in the key: size is normalized to the median value in each stack. Each panel includes data from 2 image stacks (averaging 9.7 IHCs per stack) at each cochlear location from each ear. Group sizes were as follows: *Control* (n = 6 ears, 6 animals), *1 day* (n = 6 ears, 3 animals), *1 week* (n = 6 ears, 5 animals), *1 month* (n = 6 ears, 3 animals).

**Figure 5:**
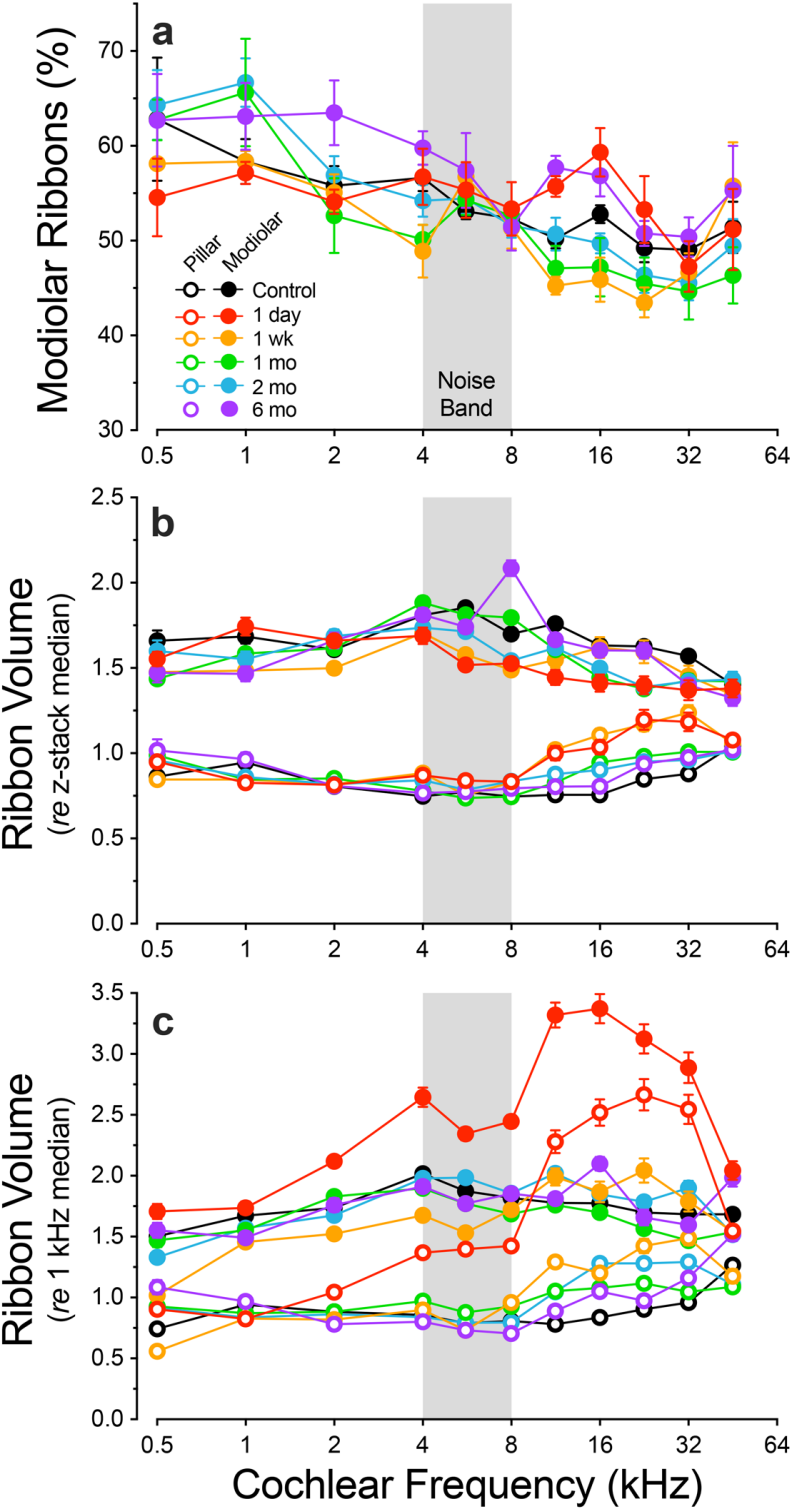
Post-exposure, there is transient hypertrophy and loss of spatial size gradient of the pre-synaptic ribbons. (**a**) Percentage of ribbons on the modiolar side of the IHCs in each post-exposure time group. (**b**,**c**) Ribbon sizes in each post-exposure time group, expressed either *re* the median for each respective z-stack (**b**), or *re* the median value in the same ear at the 1 kHz place (**c**). For all panels, data are means ± SEMs. Group sizes were as follows: *Control* (n = 13 ears, 7 animals), *1 day* (n = 6 ears, 3 animals), *1 week* (n = 6 ears, 5 animals), *1 month* (n = 6 ears, 3 animals), *2 months* (n = 6 ears, 3 animals), *6 months* (n = 6 ears, 3 animals).

The results of this spatial analysis are summarized graphically in Fig. 4. To keep the plot size reasonable, only every other cochlear location is displayed, and the 2-month survival isn’t shown, although all groups and cochlear regions were evaluated in similar fashion. With ribbon size coded by symbol color and size, the normal spatial gradient within the synapse clusters is obvious at all cochlear locations. When viewed in these zy projections, the clusters rotate progressively around the IHC basolateral pole, towards the pillar side of the HC, as the cochlear locus moves from apical (1 kHz) to basal (32 kHz) positions (Figs. 4, 5a), an observation that has not been previously noted. The physiological significance of this trend is unclear, but it is not mirrored by a shift in the spontaneous rates of ANFs as a function of cochlear frequency^18^.

The analyses from noise-exposed ears reveal several striking trends in the regions of maximal synaptic loss (e.g. 16 kHz): (1) post-exposure degradation of the modiolar-pillar gradient of ribbon size, (2) post-exposure movement of ribbons away from the IHC basolateral poles, and (3) recovery of the modiolar-pillar gradient and the normal basolateral ribbon locations by as soon as 1 month post-exposure. These observations clearly show that the noise-induced effects on synapses are not simply down- and up- regulation of staining intensity in static ANF synapses.

Although the visual impression from the graphical summary is that the modiolar-pillar ribbon-size gradient has disappeared, quantitative analysis (Fig. 5b) shows that the mean modiolar-pillar differences, though clearly attenuated, remain visible, even at 1 day post exposure and even in the region of maximum acute synaptopathy (11.3–32 kHz): p = 0.024 for modiolar vs. pillar sides (2-way ANOVA). Notably, this size gradient remains despite the increased ribbon volume observed on both pillar and modiolar halves of the IHCs 1 day following exposure (Fig. 5c). If the noise-induced synaptic loss were selective for low-SR fibers, as has been reported^35^, and if the synapses did not move around on the IHC membrane, the fraction of modiolar synapses should decrease (and possibly recover) after noise exposure. Indeed, while 1 day following exposure the changes in the modiolar fraction are not significantly different from controls in the 11–32 kHz region (p = 0.0742, 2-way ANOVA), the noise-induced reduction in the modiolar fraction is significant at 1 week and 1 month (p = 0.0042 and 0.0483, respectively). The fractions then increase until they return to normal at 2 months (p = 0.1727), and apparently overshoot by 6 months, when the fraction of modiolar ribbons exceeds that of controls (p = 0.0223). The variation in modiolar ribbon fraction across post-exposure time suggests dynamic and ongoing reorganization of ribbons following noise damage.

**Figure 6:**
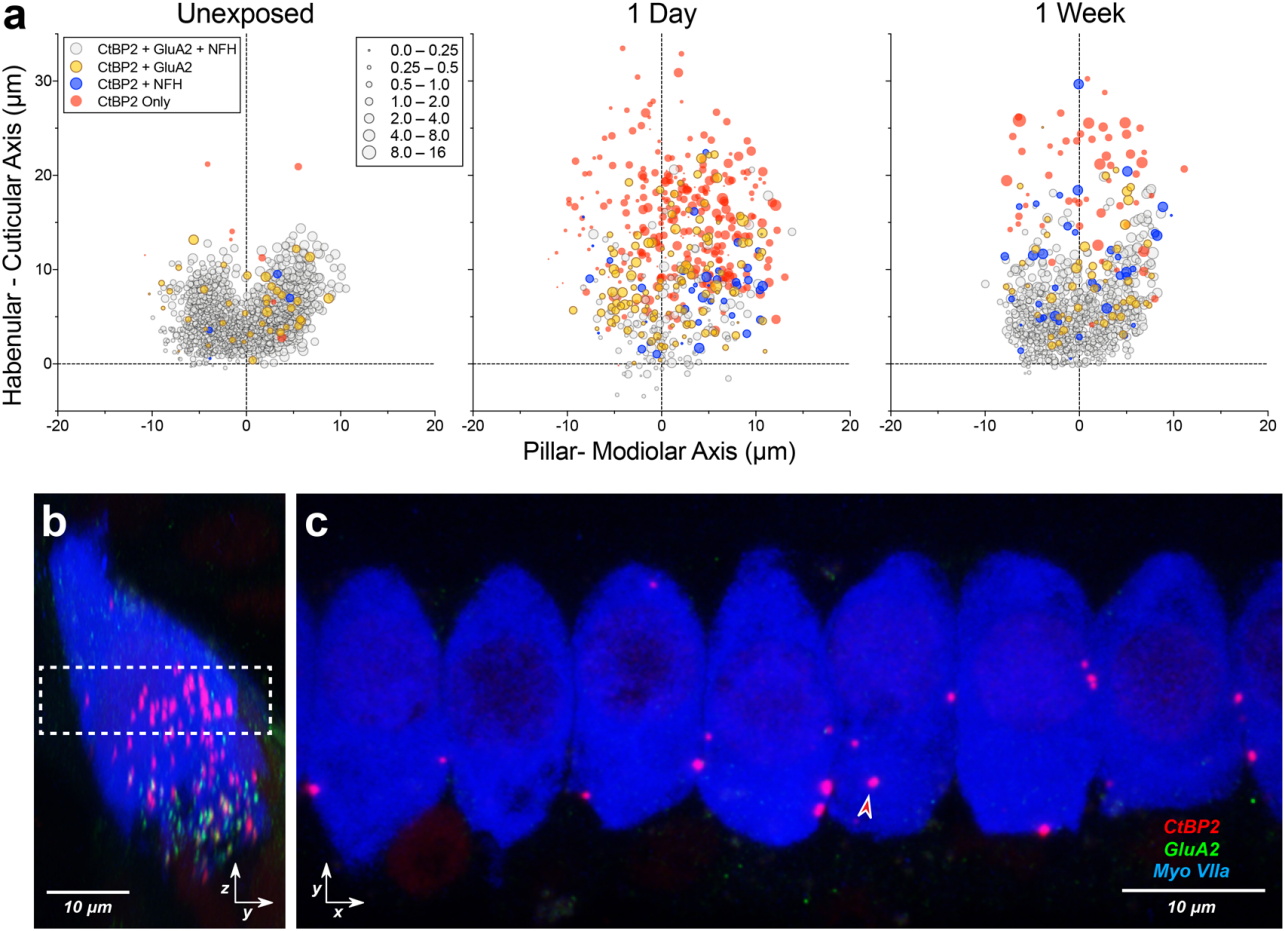
Many displaced ribbons remain paired with GluA2 puncta and/or ANF terminals, and most orphan ribbons remain near the IHC membrane. (**a**) Locations of orphan ribbons vs. synaptic ribbons for control vs. exposed ears (1–7 day post-exposure) at the 16 kHz location. Projections represent the following group sizes: *Unexposed* (n = 4 ears, 4 animals), *1 day* (n = 4 ears, 3 animals), *1 week* (n = 4 ears, 4 animals). As in Fig. 4, symbol size is proportional to ribbon volume (expressed *re* the median size for each stack), and color is used to distinguish orphan ribbons (unpaired with GluA2 puncta or ANF terminals) from ribbons paired with either GluA2 puncta or ANF terminals, as shown in the key. Pairing was assessed using custom software that creates a maximum projection of the voxel space within 2 microns of each ribbon in each z-stack^19^. (**b,c**) Confocal projections from a representative z-stack from the 16 kHz region at 1 day post-exposure, shown in the acquisition plane (x,y; **c**) and the perpendicular projection (z,y; **b**) that reveals the modiolar-pillar axis. Dashed box in (**b**) shows the z planes included in the xy projection in (**c**). Arrowhead in **c** points to the only CtBP2-positive punctum in this projection that does not appear to be at the IHC membrane.

To further probe the idea that synaptic structures are moving around the IHC membrane after noise, we assessed how many of the translocated ribbons remained synaptic, i.e. closely apposed to glutamate receptor patches, and/or ANF terminals. As shown in Fig. 6a, at both 1 day and 1 week post exposure, there are significant numbers of these paired CtBP2-GluA2 puncta (yellow-fill symbols) in exposed ears at positions farther from the basolateral pole than ever seen in normal ears. There are also numerous pre-synaptic ribbons apposed to NFH-positive terminals (blue-fill symbols), as well as complexes containing all three elements (ribbons, receptors and terminals: grey-fill symbols), all located much farther away from the IHC basolateral poles than in any unexposed ears. Even the unpaired “orphan” ribbons, that are so common at 1 day post-exposure, remain closely apposed to the IHC membrane in most cases, as shown by one representative example from the 16 kHz region (Fig. 6b,c).

**Figure 7:**
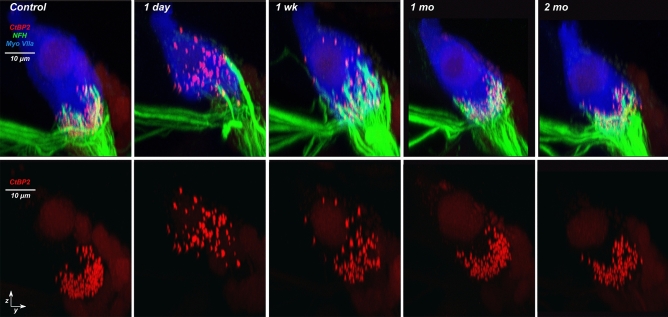
After exposure, ANF terminals transiently extend higher on the hair cell. Top row shows representative zy projections of image stacks from the 22.6 kHz region, for the post-exposure times indicated. Each re-projected stack spans roughly the same number of IHCs as in the xy projection of Fig. 2D. Bottom row shows the same image stacks, except only the CtBP2 channel is shown. Scale bar at the top left applies to all images. In some three-color images the bleed-through of the brilliant ribbon label into the NFH channel has been suppressed.

**Figure 8:**
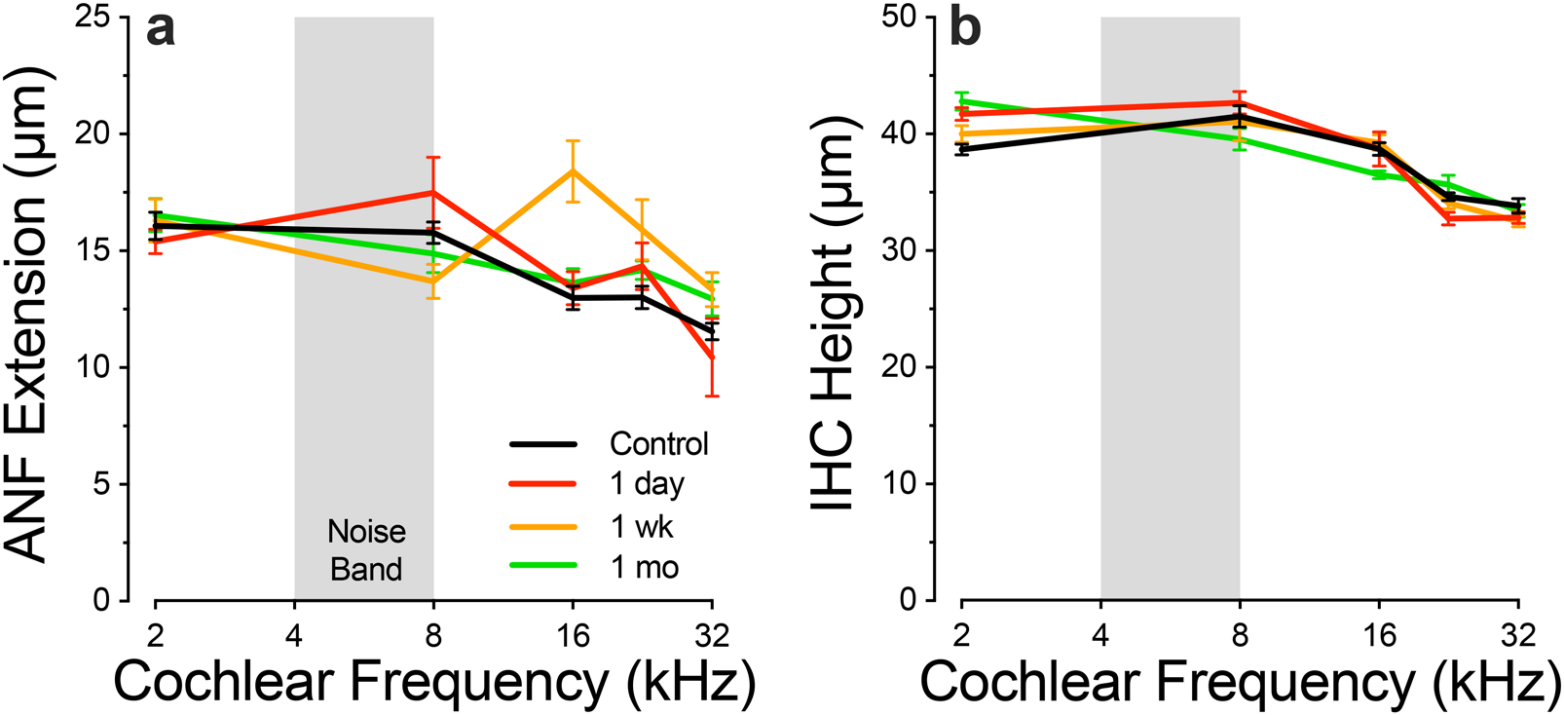
After exposure, ANFs show transient neurite extension without changes in IHC height. Data show mean values (± SEMs) for cochlear regions and survival times, as shown in the key. ANF extension (**a**) and IHC height (**b**) were measured in zy maximum projections, exactly as schematized in Fig. 2C. Each z-stack from each case produced a single value of each metric, which represented the maximum IHC height and the maximum ANF extension in that stack. Group sizes were as follows: *Control* (n = 6 ears, 6 animals), *1 day* (n = 6 ears, 3 animals), *1 week* (n = 6 ears, 5 animals), *1 month* (n = 6 ears, 3 animals).

The data in Fig. 6 suggest that synaptic complexes between ANFs and IHCs are disappearing in the first 24 h after noise exposure and then re-appearing at different locations on the IHC within the first week of recovery. Further evidence for the neurite extension that this scenario implies can be seen in the confocal projections. Images from the 22.6-kHz region (Fig. 7) show hypertrophied ANF terminals extending much farther than normal from the IHC synaptic pole at the 1-day and 1-week post-exposure survival times. This hypertrophied morphology is qualitatively different from that seen in controls and was only seen 1 day and 1 week post exposure in a blinded analysis of all z-stacks from the 16 and 22.6 kHz regions. Measures of this neurite extension across all cases (Fig. 8a) showed a highly significant elongation of ANF dendrites *re* control at 1 week post exposure (p = 0.0043, 2-way ANOVA; p = 0.036 @16 kHz, Holm-Sidak multiple comparisons), with a return to normal at 1 month (p = 0.189 2-way ANOVA; p = 0.735 @ 16 kHz, Holm-Sidak multiple comparisons). In the same z-stacks, there was no evidence for any systematic changes in the heights of the IHCs (Fig. 8b): e.g. for control vs 1 week p = 0.857 across all tested frequencies and ≥ 0.773 at 16.0 and 22.6 kHz.

## Discussion

### The phenomenon of noise-induced cochlear synaptopathy

Decades ago, ultrastructural studies of cochleas examined 24 h after acoustic overexposure noted swelling and membrane rupture of ANF terminals under IHCs^11,12^. The observations that similar histopathology could be produced by cochlear perfusion with glutamate agonists, and that noise-induced swelling could be reduced by glutamate antagonists, suggested that this pathology is a form of glutamate excitotoxicity^36^. This interpretation is further supported by recent studies of noise exposures in mice lacking the vesicular glutamate transporter in IHCs^37^, or with pharmacological blockade of calcium-permeable AMPA receptors^38^.

Because this ANF pathology disappeared in a few days, with a time course similar to the threshold recovery, it was assumed that the neural damage caused the threshold shift, and that ruptured ANF terminals and synapses regenerate^13^. However, noise-induced threshold shifts of this type are well explained by damage to the cochlear amplifier^39^, i.e. OHC function. Furthermore, cochlear (and behavioral) thresholds are extremely insensitive to loss of ANF responses^7^. Neural activation spreads along the cochlea so rapidly with increasing sound pressure that 50% loss of ANFs elevates thresholds by only a few dB^40^. Thus, many acutely damaged ANF terminals could have degenerated, despite the threshold recovery. Therefore, the observations of massive synaptopathy despite minimal threshold shift at 1 week post-exposure, as observed here (Figs. 1 vs. 3), is unsurprising since damage to the cochlear amplifier and to ANF terminals are not strictly linked.

A decade ago, our laboratory showed that noise exposures causing a large, but reversible, threshold shift also caused an immediate loss of 50% of ANF/IHC synapses^5^. The synaptopathy showed no recovery out to 8 weeks post exposure. After 2 years, degeneration of ANF cell bodies (spiral ganglion cells) matched the 50% acute loss of synapses^5^. More recent studies have corroborated the lack of post-exposure synaptic recovery in the CBA/CaJ mouse^14,15^.

In contrast, other laboratories reported significant post-exposure synaptic recovery in both mouse and guinea pig^16,27–30^, and these observations were interpreted as neural regeneration^29^. However, the disappearance and reappearance of pre- and/or post-synaptic puncta could reflect down- and subsequent up-regulation of marker expression, or internalization (and subsequent membrane relocation) of glutamate receptors^41^, rather than retraction and regeneration of ANF terminals. Indeed, we often observe post-exposure attenuation (not elimination) of synaptic puncta, and subsequent re-intensification without changes in synaptic counts. Furthermore, when pre- and post-synaptic puncta are counted independently without consideration of their spatial pairing, as in prior reports of synaptic regeneration in guinea pig^16,29^, the counts may represent orphaned ribbons (Fig. 3d) or receptor plaques rather than true functional synapses (Fig. 3a–c). Neither of these scenarios reflects true neural regeneration, i.e. destruction/retraction of unmyelinated ANF terminals, followed by neurite extension, target engagement and synaptogenesis. Because the question of whether ANF regeneration can occur spontaneously in a mature mammalian cochlea is key to our understanding of inner ear biology, and to the design of therapies for cochlear synaptopathy, we set out to re-evaluate the effects of noise on ANF synapses in the guinea pig.

### Post-exposure reorganization and regeneration of ANF synapses

Here, we replicate the post-exposure recovery of ribbon counts in guinea pigs reported previously^29^, and find that these recovered ribbons eventually re-pair with postsynaptic GluA2 receptors. Indeed, the fractional recovery at different post-exposure times is quantitatively similar in the two studies (Fig. 3 here vs. Fig. 1B in Song et al.^29^), despite differences in histological processing, postsynaptic staining and quantification, noise-exposure spectrum, and exposure level. Using identical histological techniques to those in our prior studies^5,10^, we see qualitative differences in ANF synaptic recovery after noise in mouse vs. guinea pig. Although we have previously shown dissolution and partial post-exposure recovery of ribbon-size gradients in mouse^10^, we repeatedly fail to see any recovery of synaptic counts, from 0 h to 2 years post exposure, in CBA/CaJ mice^5,14,15,42^.

Our analysis of size gradients and spatial organization of synaptic puncta show that the neuropil under the IHCs, where unmyelinated peripheral terminals of ANFs connect to IHCs, is extremely dynamic in the mature guinea pig cochlea. Synaptic complexes, including both pre- and post-synaptic active-zone proteins, are moving around the IHC membrane in the days post-exposure. These complexes climb up the IHC, away from the basolateral pole, within the first 24 h, and subsequently regain their normal positions within roughly one month (Figs. 4 and 6), as the pre-synaptic ribbons also lose and then re-establish the normal modiolar-pillar size gradient that mirrors the modiolar-pillar segregation of low- vs. high-SR ANFs in the normal ear^6,33^.

Neurofilament staining showed that some of the ectopic synaptic complexes on IHCs are also associated with ANF terminals, projecting to regions on the IHC where normally terminals are never found, and that these ectopic ANF terminals are abnormally rich in neurofilament protein (Fig. 7). Ectopic, neurofilament-rich ANF terminals are never seen in our noise-exposed mice, however they are common when either normal or noise-exposed mice are treated with a neurotrophin-expressing virus via cochlear injections that transfect IHCs^43^: in these mouse cochleas with > fourfold overexpression of NT3, there was dramatic extension of hypertrophied ANF neurites towards the IHC cuticular plates, exactly as shown here in Fig. 7. Correspondingly, there was significant rescue of synaptic counts in the mice with elevated NT3^43^.

Confocal images can never unambiguously reveal whether ANF terminals are retracting and re-sprouting; such proof must await labor-intensive serial section reconstruction at the electron-microscopic level. Nevertheless, the present results strongly suggest such retraction and regeneration is indeed happening spontaneously in the guinea pig, but not the CBA/CaJ mouse ear, unless neurotrophin levels are artificially increased.

### Possible reasons for differences in synaptic recovery

Post-exposure recovery of noise-induced cochlear synaptopathy has now been investigated in guinea pigs (Shi et al.^16^, Song et al.^29^, and present study) and two strains of mice: CBA/CaJ^5,10,14,15^ and C57Bl/6^28,30,37^. In guinea pigs, present results show recovery of synaptic counts in the most-affected region (89% loss at 1 day to only 20% loss at 6 months; Fig. 3), and a prior study from another laboratory showed ribbon recovery from 70% loss at 1 day to 25% loss at 1 month^29^. Although our prior studies of CBA/CaJ mice showed essentially no recovery out to 2 years; studies in C57Bl/6 mice from other laboratories show significant recovery in the most affected region: (1) 50% loss at 1 day to 25% loss at 2 weeks^37^, (2) 0% loss at 0 h, 50% loss at 1 day, 10% loss at 2 weeks and 0% loss at 8 weeks, in the most affected cochlear region^27^, and (3) 85% loss at 4 days to 0% loss at 2 weeks^28^.

In addition to the species/strain differences (and ignoring effects of the observable inter-laboratory differences in immunostaining signal-to-noise), other sources of difference in synaptic recovery include sex^44^ and age-at-exposure^4^. Here we studied primarily females, but saw no significant sex differences at 1 month post exposure (Fig. 1 and data not shown), and the prior guinea pig study used only males^16^. Likewise, *re* mouse studies, our CBA/CaJ work showed no difference between males and females^14^, and the C57Bl/6 studies also used both males and females^27,37^ or failed to specify^28^.

We exposed mice at 16 weeks in most of our mouse studies^5,14,15^, because CBA/CaJ become significantly less vulnerable to noise as animals age from 8 to 16 weeks^4^, and because 16 weeks is after the onset of sexual maturity (at 6–8 weeks). All the C57Bl/6 studies showing synapse recovery exposed animals at younger ages: i.e. 5 weeks^28^, 6 weeks^27^ or 8–12 weeks^37^. Here, guinea pigs were noise-exposed at 250–350 g, and those from prior studies were exposed at 300–350 g^16,29^. Birthdates are unavailable for our animals, but an internal study from our supplier (elmhilllabs.com) states that their guinea pigs are 2–3 weeks old at that weight, i.e. well before the onset of puberty, which is 12 weeks for male guinea pigs and 8 weeks for females^45^. However, one of our CBA/CaJ studies exposed animals at 6 weeks and also found no synaptic recovery out to 16 months post-exposure^42^.

Age-at-exposure could contribute to the discrepancies in the degree of spontaneous synaptic recovery, because (1) there is a prominent age-related decrease in neurotrophin-3 expression in mouse, especially in the basal half of the cochlea, where the synaptopathy is the most severe^46^, and (2) virally induced upregulation of neurotrophin-3 in our mouse model led to synaptic rescue and a phenocopy of the morphological signs of neurite extension occurring spontaneously in the guinea pig^43^. However, it is also likely that expression levels of neurotrophin-3 are species/strain-related, and subject to intra-species genetic differences. A hypothesized key role of neurotrophin expression in setting the extent of synaptic recovery in the adult ear is directly testable in a variety of ways.

## Data Availability

The datasets generated and analyzed in this study are available from the corresponding author on request.
